# Demonstrating the Potential for Dynamic Auditory Stimulation to Contribute to Motion Sickness

**DOI:** 10.1371/journal.pone.0101016

**Published:** 2014-07-01

**Authors:** Behrang Keshavarz, Lawrence J. Hettinger, Robert S. Kennedy, Jennifer L. Campos

**Affiliations:** 1 Technology Team/iDAPT, Research Department, Toronto Rehabilitation Institute, Toronto, Ontario, Canada; 2 Center for Behavioral Sciences, Liberty Mutual Research Institute for Safety, Hopkinton, Massachusetts, United States of America; 3 RSK Assessments, Inc., Orlando, Florida, United States of America; 4 Department of Psychology, University of Toronto, Toronto, Ontario, Canada; University of Salamanca- Institute for Neuroscience of Castille and Leon and Medical School, Spain

## Abstract

Auditory cues can create the illusion of self-motion (vection) in the absence of visual or physical stimulation. The present study aimed to determine whether auditory cues alone can also elicit motion sickness and how auditory cues contribute to motion sickness when added to visual motion stimuli. Twenty participants were seated in front of a curved projection display and were exposed to a virtual scene that constantly rotated around the participant's vertical axis. The virtual scene contained either visual-only, auditory-only, or a combination of corresponding visual and auditory cues. All participants performed all three conditions in a counterbalanced order. Participants tilted their heads alternately towards the right or left shoulder in all conditions during stimulus exposure in order to create pseudo-Coriolis effects and to maximize the likelihood for motion sickness. Measurements of motion sickness (onset, severity), vection (latency, strength, duration), and postural steadiness (center of pressure) were recorded. Results showed that adding auditory cues to the visual stimuli did not, on average, affect motion sickness and postural steadiness, but it did reduce vection onset times and increased vection strength compared to pure visual or pure auditory stimulation. Eighteen of the 20 participants reported at least slight motion sickness in the two conditions including visual stimuli. More interestingly, six participants also reported slight motion sickness during pure auditory stimulation and two of the six participants stopped the pure auditory test session due to motion sickness. The present study is the first to demonstrate that motion sickness may be caused by pure auditory stimulation, which we refer to as “*auditorily induced motion sickness*”.

## Introduction

Motion sickness (MS) is a well-known and widely reported malady. MS is not only a major issue among travelers (e.g., on cars, buses, trains, airplanes, or ships), but also for users of virtual environments (e.g., driving simulators or gaming systems). As real physical motion is typically absent in the latter scenarios (except for motion-based simulators), MS is often referred to as being *visually induced* in these cases (see [1] for an overview). An acute phase of MS is characterized by symptoms ranging from sensations of sudden warmth, pallor, sweating, drowsiness, and fatigue, to more severe stomach problems, increased salivation, retching, nausea, and/or vomiting [2], [3].

The precise nature of MS and its etiology is not fully understood and several theories exist (see [4] for an overview). For example, the “sensory conflict theory” [5] proposes that a mismatch between (or within) the visual, the vestibular, and/or the somatosensory senses causes MS. Based on this theory, a fixed-base simulator can cause visually induced MS due to the incongruent information delivered to the eyes (indicating self-motion, see [6] for an overview) and the vestibular and somatosensory senses (indicating a veridical, stable, and non-moving position). If the nature of the conflict is novel to the organism (i.e., no previous experience of this particular scenario), MS is possible [7]. In contrast, others postulate as a part of the “postural stability theory” that changes in the amount of postural steadiness (either reduced or increased) precede the occurrence of MS [8], [9]. A comprehensive overview of the most prominent theories explaining MS and a critical comparison of these theories is provided by [10].

None of the current theories explicitly address the role of auditory information in the genesis of MS, yet auditory cues might in fact contribute to MS in at least two ways. First, spatial sound can create illusory self-motion (vection) in the absence of visual, vestibular, or somatosensory information (see [11] for an overview). In this case, the information perceived by the auditory system contradicts the information given by other sensory modalities, thereby introducing a sensory conflict. Second, spatial sound is also known to create physical responses such as adjustments to posture [12], [13], which might influence self-motion perception and MS. Currently, it is not clear whether the strength of the response to spatially moving sound is enough to by-pass other cues to self-motion perception (i.e., visual, vestibular, somatosensory) in a way that leads to perceptual, behavioral, and physical responses such as MS or vection. Anecdotal reports indeed suggest that auditory cues can create MS symptoms (J. Lackner, personal communication, October 03, 2013), but scientific findings are non-existent (see [14], p. 33) and thus, the aim of the present study was to fill this gap.

In a recent study [15], we analyzed the effect of auditory information on vection [15]. Participants were exposed to a constantly rotating stimulus that contained either only visual, only auditory, or a combination of corresponding visual and auditory cues. These results demonstrated that auditory cues significantly increased vection strength and reduced the onset time of vection when they were added to visual cues (compared to pure visual or pure auditory stimulation). Motion sickness data was also collected as a control factor and results showed that auditory cues did not affect the level of MS. Note, however, that the primary focus in [15] was on vection and contributing factors, thus, the experimental settings were not optimized for producing and assessing MS; hence, MS-reports were generally very weak, likely resulting in a floor effect. The present study was intended to follow up on the findings by [15], but with a primary focus on introducing factors that were likely to maximize the chances of observing MS. We aimed to answer two questions: First, can auditory stimulation in the absence of visual cues elicit MS? Second, does the inclusion of dynamic auditory stimulation enhance the experience of MS when added to visual motion displays? We used the same apparatus as in [15] (e.g., laboratory, stimuli etc.), but modified the experimental parameters to be able to optimally measure MS. For instance, we prolonged the stimulus duration and we asked participants to tilt their heads to the right or left shoulder alternately while being exposed to the visually or auditorily rotating stimulus. Such head movements have previously been shown to cause *pseudo-Coriolis* sensations that can increase MS [16], [17], [18], [19]. Traditional Coriolis sensations (e.g., [20]) are experienced when the tilting of the head during full-body axis rotation causes an intra-vestibular canal-otolith mismatch that leads to severe MS. Pseudo-Coriolis sensations, on the other hand, are not related to the interactions between head rotations/vestibular feedback and physical rotations, but are induced via visual rotations. We assessed measurements of MS (severity, onset time), vection (vection strength, vection onset time, vection duration), and posture (center of pressure) using well-validated tools.

## Materials and Methods

### Participants

Twenty adults (13 female, *M_age_*  = 23.93, *SD_age_*  = 7.16; 7 male, *M_age_*  = 28.43, *SD_age_*  = 9.50) participated in this study. All participants reported that they were in a normal state of health (i.e., no vestibular disorders, no cold, no headache etc.) and had normal or corrected-to-normal vision. Participants were naïve with respect to the purpose of the study. The study protocol was reviewed and approved by the local research ethics board of the Toronto Rehabilitation Institute and participants gave written consent prior to the experiment. Additionally, the study was designed in accordance with the Declaration of Helsinki to ensure research ethics in human experimentation. Participants were compensated with $10 and were given the chance to stop stimulus exposure at any time without penalty. Note that aborting a single condition did not necessarily lead to the cessation of the entire experiment. Instead, participants were given the opportunity to rest between conditions before continuing. Half of the participants stopped stimulus exposure during at least one of the conditions, but nobody quit the whole experiment without participating in all three conditions.

### Design, apparatus, and stimuli

**Figure 1:**
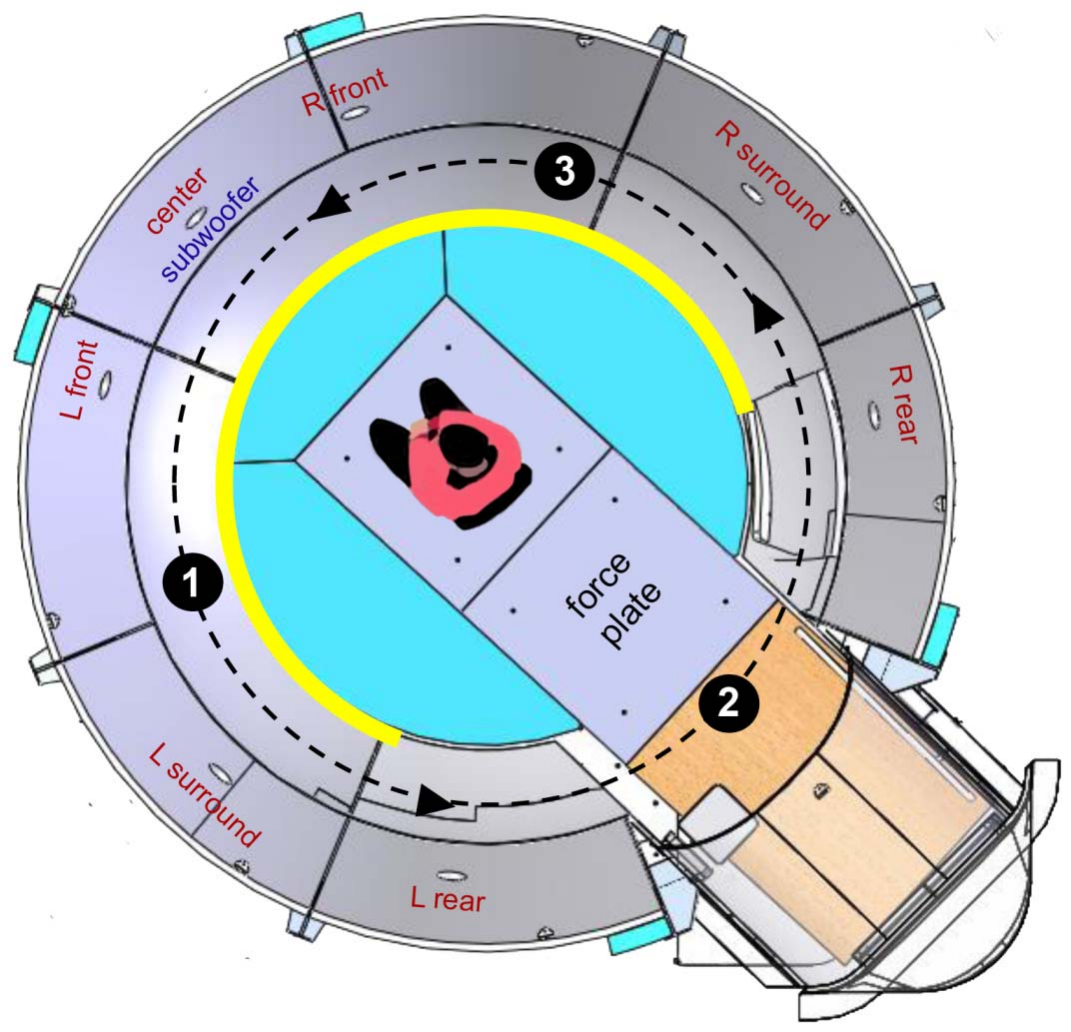
Schematic illustration of the laboratory (Bird's eye view). Participants were seated in a rotatable chair, facing the curved projection screen (thick yellow inner circle). A force plate was used to measure postural steadiness. Seven speakers (small white ovals) were arranged in a 7.1 surround sound configuration behind the projection screen, with the center channel positioned straight ahead and the other speakers aligned with respect to the center speaker at a distance of 45 degrees (left and right front), 90 degrees (left and right surround), and 135 degrees (left and right rear), respectively. The subwoofer was located at ground level below the center speaker. The black-filled circles represent the 3 sound sources used in the experiment and their spatial arrangement with respect to the participant (1 =  church bells, 2 =  streetcar, 3 =  car engine). The sound sources were synchronously rotated counterclockwise for 360° along the participants' yaw-axis (dotted black line).

A one-factorial within-subjects design including the factor stimulus condition (visual-only, auditory-only, auditory + visual) was chosen. To avoid inter-individual differences, every participant was exposed to all three conditions. Presentation order was counterbalanced to control for carryover effects. The apparatus was basically identical to the one used by [15], but the experimental procedure was modified to maximize the likelihood of MS to occur. For instance, stimulus duration was prolonged (5 min) and participants tilted their heads during stimulus exposure to the right or left shoulder alternately to generate pseudo-Coriolis effects and MS. Participants were seated in a dimly lit, dome-shaped laboratory in a rotatable chair, 100 cm in front of a curved projection screen (see Figure 1). Six projectors (Eyevis ESP-LED series with LED technology) created a visual image with a field-of-view of 240° horizontally and 120° vertically. Three-dimensional sound was provided by 7 speakers (Meyersound MP-4XP) and a subwoofer (Meyersound MP-10XP) that were arranged in a 7.1 surround sound configuration behind the projection screen. That is, the center channel was positioned straight ahead, whereas the other speakers were aligned with respect to the center speaker at a distance of 45 degrees (front left and right), 90 degrees (surround left and right), and 135 degrees (rear left and right), respectively. The subwoofer was located at ground level below the center speaker (see Figure 1). The height of the chair was fixed at 70 cm above the floor. Participants were asked to hang their feet loosely in the air without touching the ground or any other surface, as this procedure has been shown to maximize the likelihood of vection (see [21]).

The visual stimulus consisted of a VR scene of downtown Toronto that was continuously rotated for 360° along the observer's yaw axis. Based on previous findings [15], rotation speed was set to 90°/s counterclockwise and no acceleration and deceleration were used at video onset and offset, respectively. Picture resolution accounted for 6.5 arcmin/OLP and was created using customized modifications of OpenScene Graph. No components within the virtual scene were animated (e.g., no motion of cars or pedestrians). In the auditory-only and auditory + visual conditions, three different sound sources were used; church bells, the engine sound of a stationary car, and the ringing sound of a stationary streetcar/tram. The sounds were positioned in the three-dimensional virtual world, co-located with their respective visual objects. The three sound sources consisted of uncompressed monaural linear pulse code modulation Waveform Audio (.wav) files and were distributed to the speakers using the OpenAL library for rendering three-dimensional positional audio. The volume of each sound was adjusted to achieve a nominal of 75 dB and the distance between each sound source was held constant. Sound pressure level was measured at the observer's nominal head location using a sound meter application (uvex) and revealed an average of 75 dB loudness for all sound conditions. Similar to the visual scene, the sounds rotated at a speed of 90°/s in a counterclockwise direction. In the auditory-only condition, visual stimulation was excluded by blackening the projector screen and by blindfolding the participants with a sleeping mask.

### Response measures

Motion sickness was measured in three ways. First, the Fast Motion Sickness Scale (FMS) [22] was used; a verbal rating scale that was designed to monitor the time-course and the severity of MS. The FMS ranges from 0 (no sickness at all) to 20 (severe sickness). For the purpose of this study, we predefined a FMS score of 5 as the onset of MS, indicating slight, but noticeable MS. Participants were asked to stop as soon as a score of 5 was reached. Note that the FMS was designed to measure nausea and stomach awareness in particular, ignoring other MS-related symptoms such as fatigue, dizziness, or oculomotor disturbances. The main advantage of the FMS is that it is fast, intuitive, easy-to-asses, and non-intrusive. Unlike most other verbal MS rating scales, the FMS has been successfully validated and previous studies revealed high correlations between the Simulator Sickness Questionnaire (SSQ) [23] and the FMS [22]. Participants were asked to verbally judge their level of MS every 30 s during stimulus exposure. Second, we measured the time until slight but noticeable MS was reported (i.e., FMS score of 5 or higher). Once MS onset was reported, the trial was stopped to minimize the recovery time required until all symptoms subsided and to prevent any carryover or habituation effects. Third, participants were asked to fill in the SSQ [23] four times in total; once before the experiment started (baseline) and once after each of the three conditions. The SSQ is a well-established questionnaire covering 16 symptoms that are typical for MS (e.g., nausea, dizziness, fatigue etc.). Participants rate each symptom on a 4-point scale ranging from *not at all, slight, moderate* or *severe*. The SSQ provides a total score, as well as a single score for each of the three subscales *nausea, oculomotor*, and *disorientation*.

Vection onset time, vection strength, and vection duration were collected for each condition. To measure the onset time of vection, participants verbally indicated whenever they started to feel vection and the time was manually recorded by the experimenter. Vection strength data were collected after each condition using an 11-point Likert scale (0 =  non-existent, 10 =  very strong). Participants rated vection duration choosing between the categories *never, sometime, often*, or *always*.

Participants' postural steadiness was measured once before the experiment began and once after each trial, resulting in 4 postural measurements. Participants stood on a force plate (AMTI BP12001200) with closed eyes for 25 s with their feet aligned with predefined markings in a parallel position (distance between ankle bones approximately 7 cm). Postural steadiness was examined using the center of pressure (COP) calculated from the force plate. Force data were filtered using a sixth-order dual-pass Butterworth filter with a 6-Hz cutoff frequency. Matlab R2012 was used to compute COP measures of postural steadiness. COP-based measures used for analysis included variability (standard deviation) of COP excursion in the medial-lateral (ML) and anterior-posterior (AP) directions, total length of the COP path (COP length; sum of the distance between consecutive points for the 25 s dataset), and the 95% confidence area surrounding the COP (COP ellipse; defined as the area containing the center of the COP points with a 95% probability).

### Procedure and statistical analyses

**Figure 2:**
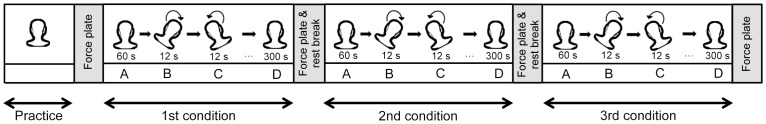
Schematic illustration of the experimental timeline and procedure. The experiment started with a practice trial to make participants familiar with the sensation of vection, followed by the first measurement of postural steadiness (force plate). At the beginning of each condition, participants remained stationary for 60 s (A), before they were verbally asked to alternately tilt their head approximately 30° towards the right or left shoulder. After tilting their head to one shoulder, participants had to hold the position for 12 s (B) before moving the head to the other side (C). This procedure was repeated until stimulus offset (5 min), or until participants reported motion sickness (i.e., FMS score of 5 or higher) (D). Postural steadiness was measured immediately after the end of each condition (force plate). Also, a rest break was provided between the conditions to allow all motion sickness symptoms to subside to normal (FMS score of 0). Participants were free to choose the duration of the rest break.

Prior to the start of the experiment, a practice trial was used to familiarize participants with the sensation of vection and with the data collection procedure. For this purpose, the auditory + visual stimulus was presented to the participants until they reported fully saturated vection (i.e., vection strength of 10). Note that the practice trial was intentionally short to avoid inducing MS and no participant complained about MS during this phase. After the practice trial, participants were exposed to all three experimental conditions in a counterbalanced order (see Figure 2). In all three conditions, participants remained stationary for the first 60 s of stimulus exposure and were told to avoid any head movements during this period. After 60 s, participants were verbally asked by the experimenter to perform pre-defined head movements, that is, they alternately tilted their heads to their right or to their left shoulder about 30°–40°. Head movements were practiced before stimulus presentation began. After tilting the head to one shoulder, participants held the position for 12 second before the experimenter verbally prompted them to move their head slowly to the other shoulder. This procedure was continuously repeated until the end of each condition (maximum of 5 min) or until MS (i.e., FMS score of 5 or higher) was reported. A rest break was provided between two consecutive conditions. Participants were free to choose the duration of the rest break and the experiment continued with the next condition once all symptoms subsided to normal (i.e., FMS score of 0).

During stimulus exposure, participants had to verbally judge their level of MS by choosing a single score from the FMS every 30 s. Whenever a FMS score of 5 or higher was reported by the participants, the stimulus was stopped immediately and participants were provided with a rest break until they indicated that they had completely recovered from any MS symptoms. Additionally, participants were asked to verbally indicate whenever they started to feel vection and vection strength was collected verbally after each condition. The first measure of postural steadiness was conducted after the practice trial and acted as a reference with respect to the subsequent measurements. After each condition, postural steadiness data were collected immediately after stimulus offset using the same procedure. After the final condition, participants were debriefed and given additional recovery time before leaving the experimenter's care.

For all statistical analyses the Statistical Package for Social Sciences (SPSS v.21, IBM) was used. A priori significance level was set to α = 0.05. Note that statistical analyses revealed no effect of trial order for any of the dependent measurements, indicating that counterbalancing the trial order successfully prevented carryover or habituation effects.

## Results

### Motion sickness data

#### Motion sickness onset time

MS onset time was defined as the time between when the stimulus was initiated until the time at which participants first reported an FMS score of 5 or higher. Note that for conditions during which sickness was not reported, MS onset time was set to the maximum of 300 s. Averaged MS onset time was shortest in the visual-only condition (*M* = 203.17, *SD* = 97.96), followed by the combined auditory + visual condition (*M* = 210.47, *SD* = 100.14), and the auditory-only condition (*M* = 289.33, *SD* = 36.15). A one-factorial repeated-measures (rm) ANOVA (Huynh-Feldt corrected) including the factor stimulus condition (visual-only, auditory-only, auditory + visual) was calculated. Note that the normality assumption regarding the FMS peak scores was violated. However, ANOVAs have been shown to be robust against violations of normality when group sizes are not small (see [24]). The rmANOVA revealed a significant effect of stimulus condition, *F*(2, 38) = 11.18, *p<*.001, η^2^ = .37, ε = .84. Simple contrast comparisons demonstrated significantly reduced MS onset times for both the combined auditory + visual condition, *F*(1, 19) = 12.38, *p = *.002, η^2^ = .39, and the visual-only group, *F*(1, 19) = 13.99, *p = *.001, η^2^ = .42, compared to the auditory-only condition. No difference between the combined auditory + visual and the visual-only condition was observed, *F*(1, 19) = 2.98, *p* = .112, η^2^ = .21.

#### FMS scores

**Figure 3:**
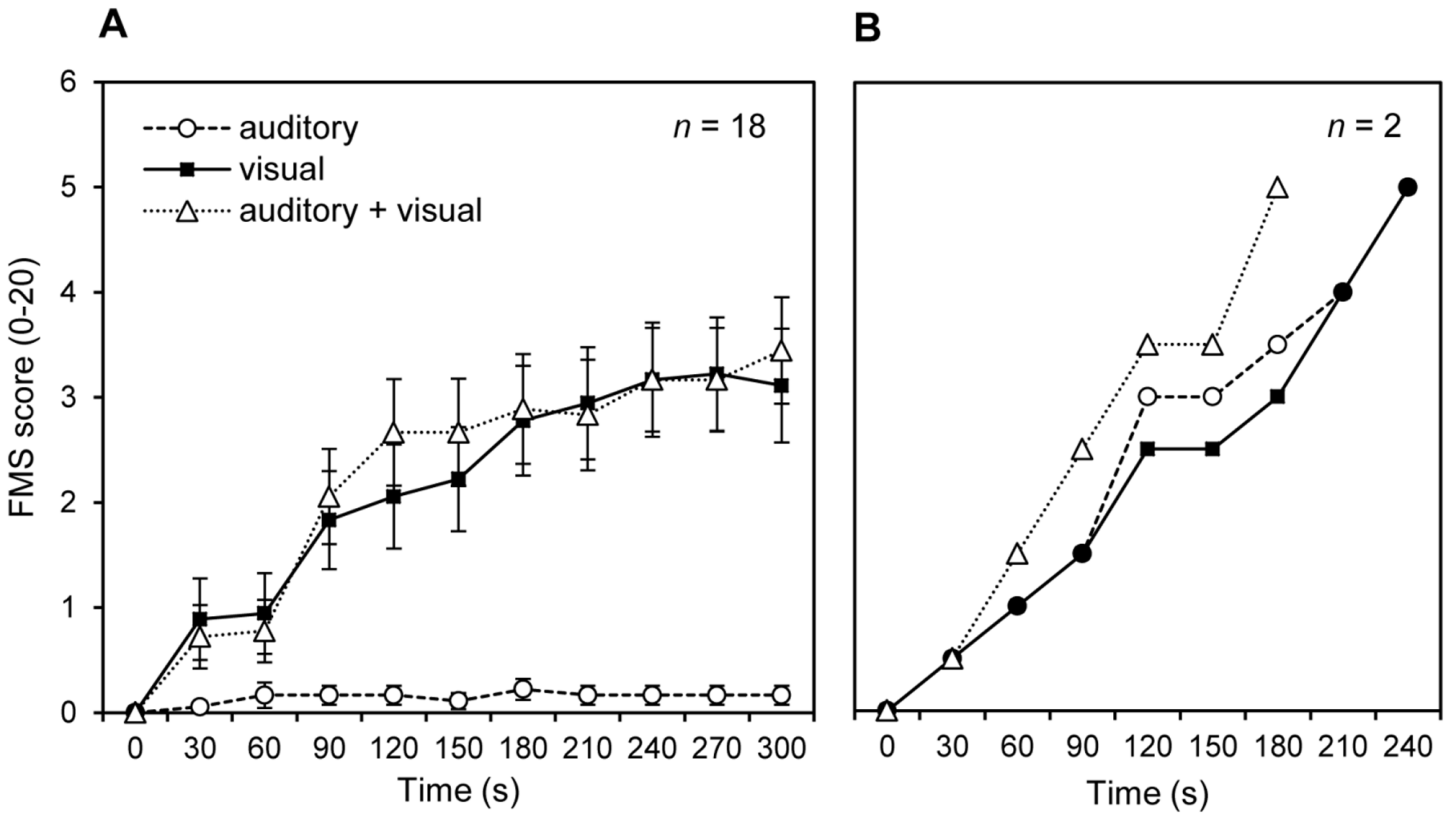
Time-course of motion sickness for all three stimulus conditions. The left panel (A) shows averaged motion sickness scores (FMS) scores for all 18 participants minute-by-minute, whereas the right panel (B) shows averaged FMS scores for the two participants who reported auditorily induced MS. Note that a FMS score of 5 was predefined as slight but noticeable MS and was selected as cut-off to stop stimulus exposure. Error bars indicate the standard error of mean.

Table 1 shows the distribution of the peak FMS scores (i.e., the highest FMS score reported during stimulus presentation) for each stimulus condition. The time-course of the FMS scores is illustrated in Figure 3. Strongest MS was reported in the auditory + visual condition (*M* = 3.75, SD = 1.86), followed by the visual-only condition (*M* = 3.45, SD = 2.16) and the auditory-only condition (*M* = 0.85, SD = 1.53). One-sampled t-tests tested against zero revealed significantly increased MS scores after stimulus exposure compared to baseline for all three conditions, including auditory + visual, *t*(19) = 9.02, *p*<.001, visual-only, *t*(19) = 7.13, *p*<.001, and auditory-only, *t*(19) = 2.48, *p = *.023. A one-factorial rmANOVA including stimulus condition (visual-only, auditory-only, auditory + visual) was calculated for the peak FMS scores. A significant effect of stimulus condition was observed, *F*(2, 38) = 25.34, *p*<.001, η^2^ = .57, ε = .68. As revealed by simple contrast comparisons, averaged FMS scores were higher in the auditory + visual condition compared to the auditory-only condition, *F*(1, 19) = 32.02, *p*<.001, η^2^ = .63, but not compared to the visual-only condition, *F*(1, 19) = 1.69, *p = *.209, η^2^ = .08. The visual-only condition showed higher FMS scores than the auditory-only condition, *F*(1, 19) = 23.61, *p*<.001, η^2^ = .55. Two participants reported a FMS score of 5 during pure auditory stimulation and stopped stimulus exposure. For both participants, the auditory-only condition was presented last, following the two other conditions. Note that the 5 other participants, who also performed the auditory-only condition last, did not report MS.

**Table 1:** Distribution of motion sickness severity (peak FMS score) and vection strength ratings for each stimulus condition (in percent).

Score	Response measure					
	Vection (0–10)			FMS (0–20)		
	Auditory + visual	Visual- only	Auditory-only	Auditory + visual	Visual-only	Auditory-only
0	—	—	65%	10%	20%	60%
1	—	—	10%	10%	10%	25%
2	—	5%	15%	5%	—	5%
3	—	5%	5%	5%	5%	—
4	—	15%	—	10%	5%	—
5	5%	—	—	60%	60%	10%
6	10%	15%	—	—	—	—
7	25%	35%	—	—	—	—
8	25%	10%	—	—	—	—
9	20%	—	—	—	—	—
10	15%	15%	5%	—	—	—
Total	100%	100%	100%	100%	100%	100

#### SSQ scores

**Figure 4:**
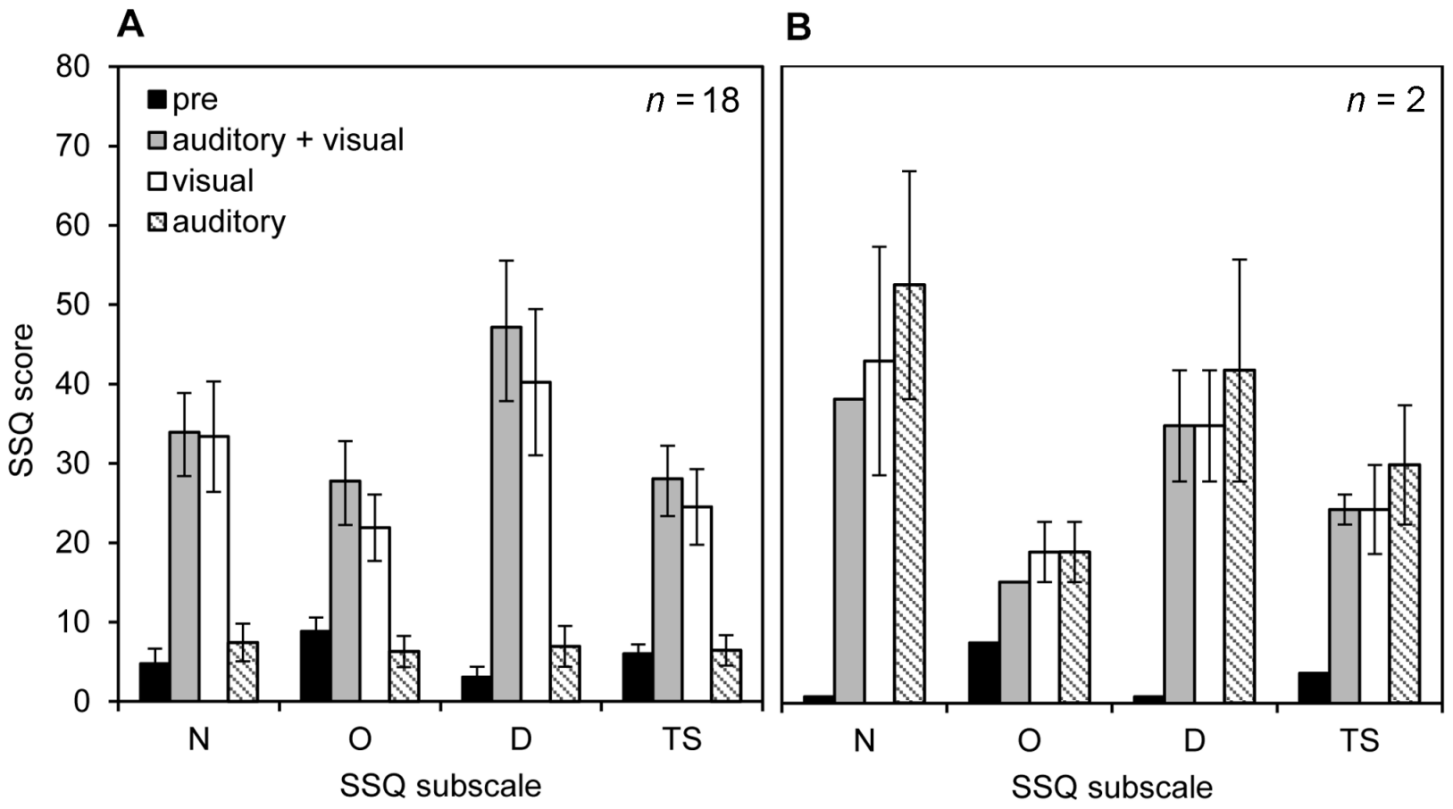
Simulator Sickness Questionnaire scores for each condition. Mean scores for the Simulator Sickness Questionnaire (SSQ) and the subscales nausea (N), oculomotor (O), disorientation (D), and total-score (TS) collected prior to the start of the experimental trials (pre) and after each stimulus condition (visual-only, auditory-only, auditory + visual). The left panel (A) shows the averaged results for all 18 participants, whereas the right panel (B) shows the results for the two participants who reported auditorily induced MS. Error bars indicate the standard error of mean.

The mean SSQ scores for each condition are shown in Figure 4. A one-factorial rmANOVA including stimulus condition (pre-exposure, visual-only, auditory-only, auditory + visual) was calculated for the SSQ subscales nausea (N), oculomotor (O), disorientation (D), and the total score (TS). Results for the rmANOVA and simple contrast comparison are given in Table 2.

**Table 2:** Repeated-measures ANOVA (Huynh-Feldt corrected) for all SSQ sub-scores including contrast comparisons for stimulus condition (visual-only vs. auditory-only vs. auditory + visual).

SSQ subscale	Comparison	*df1, df2*	*F*	*p*	η^2^	ε
**Nausea (SSQ-N)**	Main effect	3, 57	14.27	<.001**	.43	.81
	PRE vs. AV	1, 19	31.28	<.001**	.62	
	PRE vs. V	1, 19	20.43	<.001**	.52	
	PRE vs. A	1, 19	3.51	.076	.16	
	AV vs. V	1, 19	0.00	1.0	.00	
	AV vs. A	1, 19	15.83	.001**	.46	
	V vs. A	1, 19	9.35	.006**	.16	
**Oculomotor (SSQ-O)**	Main effect	3, 57	12.54	<.001**	.40	.70
	PRE vs. AV	1, 19	13.76	.001**	.42	
	PRE vs. V	1, 19	13.36	.002**	.41	
	PRE vs. A	1, 19	0.28	.603	.02	
	AV vs. V	1, 19	2.28	.148	.11	
	AV vs. A	1, 19	17.88	<.001**	.48	
	V vs. A	1, 19	14.05	.001**	.43	
**Disorientation (SSQ-D)**	Main effect	3, 57	16.68	<.001**	.47	.61
	PRE vs. AV	1, 19	26.89	<.001**	.59	
	PRE vs. V	1, 19	21.43	<.001**	.53	
	PRE vs. A	1, 19	4.61	.045*	.20	
	AV vs. V	1, 19	1.35	.259	.07	
	AV vs. A	1, 19	16.82	.001**	.47	
	V vs. A	1, 19	11.19	.003**	.37	
**Total-score (SSQ-TS)**	Main effect	3, 57	16.34	<.001**	.46	.76
	PRE vs. AV	1, 19	28.24	<.001**	.60	
	PRE vs. V	1, 19	19.96	<.001**	.51	
	PRE vs. A	1, 19	1.42	.248	.07	
	AV vs. V	1, 19	1.09	.310	.05	
	AV vs. A	1, 19	20.15	<.001**	.52	
	V vs. A	1, 19	11.74	.003**	.38	

** highly significant, * significant. AV  =  auditory + visual, A =  auditory-only, V =  visual-only

A one-factorial rmANOVA including the factor trial order (first, second, third) was run to analyze potential effects of the trials' presentation order. No significant results for trial order were observed for MS onset time, peak FMS score, the three SSQ subscales, or the SSQ total score (ranging from *p = *.31 to *p* = .93).

### Vection data

#### Vection strength

The distribution of vection ratings for each stimulus condition is given in Table 1. Averaged vection strength scores are given in Table 3. A one-factorial rmANOVA including the factor stimulus condition (visual-only, auditory-only, auditory + visual) demonstrated a significant effect, *F*(2, 38) = 131.96, *p*<.001, η^2^ = .87. Simple contrast comparisons indicated strongest vection in the combined auditory + visual condition compared to the visual-only, *F*(1, 19) = 15.26, *p* = .001, η^2^ = .45, and the auditory-only condition, *F*(1, 19) = 210.89, *p*<.001, η^2^ = .92. Also, the visual-only condition resulted in stronger vection compared to the auditory-only condition, *F*(1, 19) = 121.43, *p*<.001, η^2^ = .87.

**Table 3:** Mean (SD) vection scores (strength, onset time, duration) for all three stimulus conditions.

Vection measurement	Stimulus condition		
	Auditory + visual	Visual-only	Auditory-only
	M (SD)	M (SD)	M (SD)
Strength (0–10)	7.90 (1.41)	6.50 (2.24)	1.05 (2.31)
Onset time (s)	6.69 (3.20)	10.98 (10.43)	187.90 (138.89)
Duration (0–3)	2.80 (0.41)	2.55 (0.69)	0.45 (0.76)

#### Vection onset time

Averaged vection onset times are given in Table 3. Note that in the absence of reported vection, onset time was set to the maximum of 300 s. A one-factorial rmANOVA (Huynh-Feldt corrected) including the factor stimulus condition (visual-only, auditory-only, auditory + visual) revealed a significant effect, *F*(2, 38) = 43.25, *p*<.001, η^2^ = .70, ε = .50. Simple contrast comparisons showed significantly reduced onset times for the combined auditory + visual condition compared to the visual-only, *F*(1, 19) = 5.16, *p* = .035, η^2^ = .21, and compared to the auditory-only condition, *F*(1, 19) = 43.99, *p*<.001, η^2^ = .70. Also, the visual-only group showed reduced onset times compared to the auditory-only group, *F*(1,19) = 43.62, *p*<.001, η^2^ = .69.

Again, a one-factorial rmANOVA including trial order (first, second, third) was performed to analyze potential effects of the trials' presentation order. No significant results for trial order were observed for vection strength and vection onset time (*p*'s >.63).

#### Vection duration

Averaged vection duration for each condition is highlighted in Table 3. Participants rated the duration they felt vection during stimulus exposure on a scale from 0 (*never*) to 3 (*always*). As vection duration was measured using an ordinal scale, a non-parametric test (Friedman) was chosen and revealed a significant effect of stimulus condition, χ^2^(2) = 35.23, p<.001. Pairwise comparisons (Wilcoxon) showed significantly less vection in the auditory-only condition compared to the visual-only condition, *Z* = −3.90, *p*<.001, and the combined auditory + visual condition, *Z* = −3.92, *p*<.001, respectively. No difference was observed between the visual-only and the auditory + visual condition, *Z* = −1.52, *p* = .129.

A non-parametric test (Friedman) comparing across trial order (first, second, third) was performed to analyze potential effects of the trials' presentation order. No significant results for trial order were observed for vection duration (*p = *.95).

### Postural steadiness data

A one-factorial rmANOVA including stimulus condition (pre-exposure, visual-only, auditory-only, auditory + visual) was calculated for COP-based measures in the medial-lateral and anterior-posterior directions, total length of the COP path, and the 95% confidence area surrounding the COP. A significant effect of condition was observed only for the total length, *F*(3,48) = 5.068, *p = *.004, η^2^ = .241, ε = 1.000. Simple contrast comparisons showed significantly decreased total sway length pre-exposure compared to each of the three conditions, including combined auditory + visual, *F*(1,16) = 6.87, *p = *.019, η^2^ = .300, visual-only, *F*(1,16) = 8.32, *p = *.011, η^2^ = .342, and auditory-only condition, *F*(1,17) = 5.82, *p = *.028, η^2^ = .267. The three conditions did not vary significantly from each other. No significant results were observed for any of the other COP data. Additionally, we separated our sample into participants who reported sickness while being exposed to the stimulus and those who did not report sickness. This was done separately for each condition and *t* test comparisons showed no difference between the sick and the non-sick group regarding any of the postural steadiness parameters.

Bivariate Pearson's correlations were calculated for MS severity (measured by all SSQ scores and the FMS) and the postural steadiness measurements prior to the start of the experiment and after each condition. Correlations varied widely (from *r* = .479 to *r* = −.479), however, none of the correlations were significant (from *p* = .051 to *p* = .941).

## Discussion

The present study aimed to determine the role of auditory cues—either in addition to, or in the absence of corresponding visual information—on the experience of MS when experimental parameters were chosen to maximize the likelihood of MS to occur. Our results showed that MS was generally not affected by the inclusion of auditory cues. Specifically, MS severity and the onset of MS did not change when auditory cues were added to visual stimulation.

However, in the absence of visual information, pure auditory stimulation created weak, but significantly increased MS compared to baseline. Further, two participants reported MS during pure auditory stimulation and had to stop stimulus exposure. Despite the overall weak effect of pure auditory stimulation on MS, the present study demonstrated that MS can be elicited by pure auditory stimulation under certain circumstances and in certain individuals. In the following, we will discuss the role of auditory cues for MS in general and the unique finding of *auditorily induced MS* in particular.

### Auditorily induced motion sickness

Research focusing on understanding the relationship between auditory vection and MS is, to our knowledge, almost nonexistent ([14]; but see [15]). Thus, we believe that the present study was the first to empirically demonstrate that pure auditory stimulation may cause MS in some participants. We can think of two explanations for the occurrence of auditorily induced MS, including sensory conflict and eye-movements as potential explanations. Support for sensory conflict is given by the fact that the only two participants who suffered from auditorily induced MS also experienced auditory vection. Accordingly, auditory cues might have indicated self-motion and might have contradicted the information delivered by vestibular and somatosensory senses (no conflict between the auditory and the visual system was present, as visual information was eliminated by blindfolding participants during stimulus), which may have resulted in MS. If this is true, the traditional sensory conflict hypothesis—as described by Reason and others—possibly needs to be extended. Specifically, our findings indicate that not only the visual, vestibular, and somatosensory senses can be involved in the genesis of MS, but also that the auditory system might contributes to this conflict. The second possible explanation includes the role of eye-movements. Others studies [25], [26], [27] have reported that a rotating sound source elicits audiokinetic nystagmus in some participants, similar to optokinetic nystagmus elicited by optokinetic drums (but see [28] for contradicting results). Additionally, Ebenholtz and colleagues' [29], [30] eye-movement theory of MS specifies that optokinetic nystagmus innervates the vagal nerve by stimulating cells within the vestibular nuclei and that such innervations can elicit MS-typical outcomes such as nausea or emesis. Thus, it is possible that the auditory vection reported by the two subjects in our study was accompanied by auditorily induced nystagmus that might have elicited MS-like feelings. As we did not record eye-movement data, we cannot determine the impact of eye-movements on auditorily induced MS.

Note that only two of our 20 participants reported auditorily induced MS, whereas the vast majority of participants did not experience sickness. Several questions arise from this finding related to the clearly observable individual differences. The level of vection experienced during stimulation might be one crucial factor, as the two participants who reported auditorily induced MS were the only ones who reported moderate to strong auditory vection (score of 3 and 10, respectively). If vection is a prerequisite for MS to occur, it is not surprising that other participants did not experienced MS given that the average level of auditory vection was rather weak in the present study. To address this question more precisely, it will be important to explore other factors that might differentiate those two individuals from other participants, such as their hearing ability, the sensitivity of their other sensory systems, their past experiences, their education, training, and profession, or other cognitive and perceptual characteristics.

It is important to note that stimulus exposure was stopped as soon as a FMS score of 5 was reached, which is a conservative measure of MS (slight). We deliberately chose to stop stimulus exposure at this early stage to prevent severe MS, which might have resulted in prolonged rest breaks between the conditions and/or a higher number of participants who would have refused to continue with the experiment. This low cut-off score also helped to minimize carryover and habituation. Even with this very conservative definition, our results clearly provide evidence for the existence of auditorily induced MS. This assumption is also supported by the SSQ scores that indicated increased MS after stimulus presentation. We believe that its strength may be even more apparent with prolonged stimulus exposure. Nevertheless, future studies should further define the nature and the potential strength of auditorily induced MS.

It should also be noted that we did not measure the effect of simply making head movements or being blindfolded on MS and vection ratings (i.e. in the complete absence of auditory or visual stimulation). Thus, we cannot fully exclude the possibility that head movements or blindfolding might have led to increased MS.

### Multisensory cues, vection, and motion sickness

The relationship between vection and visually induced MS is still a matter of debate [31], [32], [33], [34], [35]. In the present study, all participants who reported visually induced MS also reported vection. However, the issue of whether visually induced MS can be elicited in the absence of vection remains unknown. Further research trying to generate visually induced MS without eliciting vection is essential to clearly address this question.

The vection-enhancing effect of sound when combined with visual stimulation has been reported before [15], [27], [36], [37]. In contrast, the role of combined visual and auditory cues with respect to MS has been largely neglected in the current literature and we are aware of only a few studies examining auditory-visual interactions in the context of MS [38], [39]. For instance, [40] presented a real-world video recorded during a bicycle ride, either excluding or including the corresponding background sounds (e.g., street noises). Similar to the findings of the current study, results showed no differences between the two groups, indicating that the addition of ecologically valid background sounds did not measurably affect the occurrence or the severity of MS beyond that induced by visual stimuli alone.

Note that in the present study participants were exposed to congruent auditory and visual cues, but were not introduced to a scenario where auditory and visual cues were both present but did not match each other (e.g., auditory and visual cues moving in opposite directions). Presenting conflicting cues might help to quantify individual effects of different sensory modalities that might play a role in the genesis of MS. The basic mechanisms of multisensory integration during auditory-visual interactions have been extensively described in the past (see [41] for an overview). During circumstances under which visual and auditory information are not in accordance, misperceptions or altered perceptions may occur, such as the ventriloquist effect (i.e., localization of auditory cues is changed by non-corresponding visual cues; see [42]), the McGurk effect (i.e., altered interpretation of spoken letters by non-congruent visual and auditory cues; see [43]), or the audiogyral illusion (i.e., misperception of auditory localization during rotation; see [44]). Interestingly, [45] showed that the audiogyral illusion correlates highly with the somatogyral illusion and that longer audiogyral illusions are significantly related to greater MS susceptibility. The authors discuss the role of the central vestibular velocity storage time constant as a crucial mechanism mediating the relationship between MS susceptibility and the audiogyral and somatogyral illusion, respectively. Except for the study by [45], no other study has focused on MS as a result of audio-visual, audio-vestibular, or audio-somatosensory mismatches. The question of whether auditory cues are powerful enough to compete with visual, vestibular, or somatosensory cues in the context of self-motion perception to the extent that mismatches including the auditory sense might result in MS has yet to be addressed.

### Postural steadiness, vection, and MS

We found a significant increase in the total path length of postural steadiness after each condition compared to baseline, indicating that participants' postural steadiness decreased after each condition compared to the beginning of the test session. As each trial caused at least slight MS, this finding is in line with the postural instability hypothesis [8], [9] and matches previous findings reporting that participants experiencing MS also showed a decrease in postural steadiness [46], [47]. In contrast, our results differ from those reported by [15], who did not find significant changes in postural steadiness after exposing their participants to a similar rotating stimulus, but with reduced duration and without head movements. This disparity is possibly due to the fact that MS was almost non-existent in [15], whereas all of the conditions in the current study induced at least slight MS. Hence, comparing across the results of these two studies, it would appear that aftereffects in postural steadiness may mainly be associated with experienced MS, rather than experienced vection.

The present study also shows that pure auditory stimulation can evoke subsequent postural responses, supporting the assumption that binaural sound cues can contribute to balance and orientation responses (e.g., [48]). In general, the vestibular system is known to be directly activated by certain auditory stimulation such as monoaural, high intensity, low-frequent sounds (see [49] and references therein), resulting in a vestibulo-postural reflex of the lower limbs. However, it seems unlikely that a direct pathway between the auditory stimulation and the vestibular response explains our results best, as the auditory stimuli used in the present study are not in the range of sounds that provoke a vestibulo-postural reflex. Instead, we believe that the rotating sounds—similar to the visual stimuli—caused an increase in disorientation, which was mirrored in the postural responses. The significant increase of the SSQ disorientation sub-score following the pure auditory stimulation strengthens this assumption.

None of the COP measurements revealed significant differences between the three sensory conditions (visual-only vs. auditory-only vs. auditory + visual). Also, no difference in postural steadiness was found between participants who reported MS compared to those who did not report MS during stimulus exposure, contradicting previous findings [46], [47]. This rather surprising result might be traced back to the fact that stimulus exposure was stopped as soon as MS onset was reported and therefore strong MS was prevented. Changes in postural steadiness might have been more apparent if MS severity was higher in the sick group compared to the non-sick group. Also note that we only collected COP data after stimulus exposure and not during stimulus exposure. Hence, the null-effect of stimulus condition found in our study might be due to a quick recalibration of the perceptual-motor system after stimulus offset.

## Conclusion

The present study found that MS was not affected by the inclusion of corresponding auditory information such that visual-only and combined auditory + visual cues elicited comparable sickness results. In contrast, auditory cues increased the level of vection when they were added to the corresponding visual stimulus, whereas vection elicited by pure auditory stimulation was rather weak. Most interestingly, MS symptoms induced by pure auditory stimulation were observed in a sub-set of participants, demonstrating the existence of auditorily induced MS.
